# Phantom limb therapy improves cortical efficiency of the sensorimotor network in a targeted muscle reinnervation amputee: a case report

**DOI:** 10.3389/fnins.2023.1130050

**Published:** 2023-05-10

**Authors:** Jordan A. Borrell, Arun Karumattu Manattu, Christopher Copeland, Kaitlin Fraser, Andrew D’Ovidio, Zach Granatowicz, Alex C. Lesiak, Sean C. Figy, Jorge M. Zuniga

**Affiliations:** ^1^Department of Biomechanics, University of Nebraska at Omaha, Omaha, NE, United States; ^2^Center for Biomedical Rehabilitation and Manufacturing, University of Nebraska at Omaha, Omaha, NE, United States; ^3^Orthopedic Surgery, OrthoNebraska Hospital, Omaha, NE, United States; ^4^Plastic and Reconstructive Surgery, University of Nebraska Medical Center, Omaha, NE, United States

**Keywords:** functional near infrared spectroscopy, amputation – rehabilitation, targeted muscle reinnervation (TMR), phantom limb movements, hemodynamic responses, neural plasticity

## Abstract

Targeted muscle reinnervation (TMR) surgery involves the coaptation of amputated nerves to nearby motor nerve branches with the purpose of reclosing the neuromuscular loop in order to reduce phantom limb pain. The purpose of this case study was to create a phantom limb therapy protocol for an amputee after undergoing TMR surgery, where the four main nerves of his right arm were reinnervated into the chest muscles. The goal of this phantom limb therapy was to further strengthen these newly formed neuromuscular closed loops. The case participant (male, 21- years of age, height = 5′8″ and weight = 134 lbs) presented 1- year after a trans-humeral amputation of the right arm along with TMR surgery and participated in phantom limb therapy for 3 months. Data collections for the subject occurred every 2 weeks for 3 months. During the data collections, the subject performed various movements of the phantom and intact limb specific to each reinnervated nerve and a gross manual dexterity task (Box and Block Test) while measuring brain activity and recording qualitative feedback from the subject. The results demonstrated that phantom limb therapy produced significant changes of cortical activity, reduced fatigue, fluctuation in phantom pain, improved limb synchronization, increased sensory sensation, and decreased correlation strength between intra-hemispheric and inter-hemispheric channels. These results suggest an overall improved cortical efficiency of the sensorimotor network. These results add to the growing knowledge of cortical reorganization after TMR surgery, which is becoming more common to aid in the recovery after amputation. More importantly, the results of this study suggest that the phantom limb therapy may have accelerated the decoupling process, which provides direct clinical benefits to the patient such as reduced fatigue and improved limb synchronization.

## 1. Introduction

The loss of a limb leads to plasticity in the sensorimotor cortex that is frequently accompanied by the vivid experience that the missing limb is still present and can be moved at will (i.e., phantom limb movement) as well as experience phantom sensations (i.e., pain, touch, etc.) (Flor et al., 1995; Kooijman et al., 2000). This is an indication that the central pathways associated with the amputated peripheral nerves retain at least some sensory and motor function and that the neural networks associated with the missing limb still exist. It has been shown that chronic amputees can still retain significant residual connectivity and function for many years after limb amputation (Dhillon et al., 2004). Numerous reports of cortical reorganization after amputations in humans have shown that neighboring cortical areas of intact body parts expand into cortical areas previously devoted to an injured or missing limb (Elbert et al., 1994; Weiss et al., 2000; Karl et al., 2001). However, much of this work has been conducted in amputees whose nerves remain severed and left in the stump of the arm after amputation (i.e., indicating an open loop system).

Recently, new surgical strategies have been conducted to preserve the severed peripheral nerves by reinnervating them into a nearby muscle (i.e., creating a new closed loop system) with the goal of reducing phantom pain, which might be directly related to the plasticity occurring within the de-afferented area of the cortex. This new type of reinnervation surgery, targeted muscle reinnervation (TMR), involves the coaptation of amputated nerves to nearby redundant motor nerve branches (Mioton and Dumanian, 2018). A successful TMR surgery allows voluntary motor control signals that use to activate muscles in the amputated limb to activate these newly reinnervated muscles thus creating a new closed loop system. The long-term surgical goal of TMR is to create new and reliable electromyography (EMG) signals in amputees to provide greater control over myoelectric prostheses. Residual upper limb nerves in the stump are paired with muscles that retain contractility but no longer have a biomechanical function. Following successful neurotization, these muscles biologically amplify nerves signals and serve as a conduit to the skin surface, thus providing new, discrete EMG signals that can be utilized for prosthetic control. The goal is to harness the original function of the severed nerve to control a specific movement of the prosthetic limb.

After the regular physical therapy after amputation, however, it is uncertain if the de-afferented sensorimotor cortex remains preserved due to this new closed-loop feedback approach. A hand transplantation study has shown a complete reversal of this cortical reorganization where the transplanted hands expand back into the deprived cortical area (Giraux et al., 2001). Additionally, it is uncertain if the reinnervated muscle produce a stable electromyographic (EMG) signal or if they need additional training. Thus, a unique phantom limb therapy was created for the subject with the goal of improving, preserving, and/or creating new, functional neural networks. This method was chosen over other methods, such as virtual reality, as this subject did not have access to additional means of physical therapy and rehabilitation past the normal rehabilitation amputees receive after losing a limb. The overall goals for this project were to aid this subject and test a unique phantom limb paradigm that could be performed at home and with no additional cost to the subject. The results shown could potentially help in future rehabilitation paradigms for TMR patients as well as in the design and control of an electrically-drive prosthesis for TMR patients.

## 2. Case description

### 2.1. Case participant

**Figure 1:**
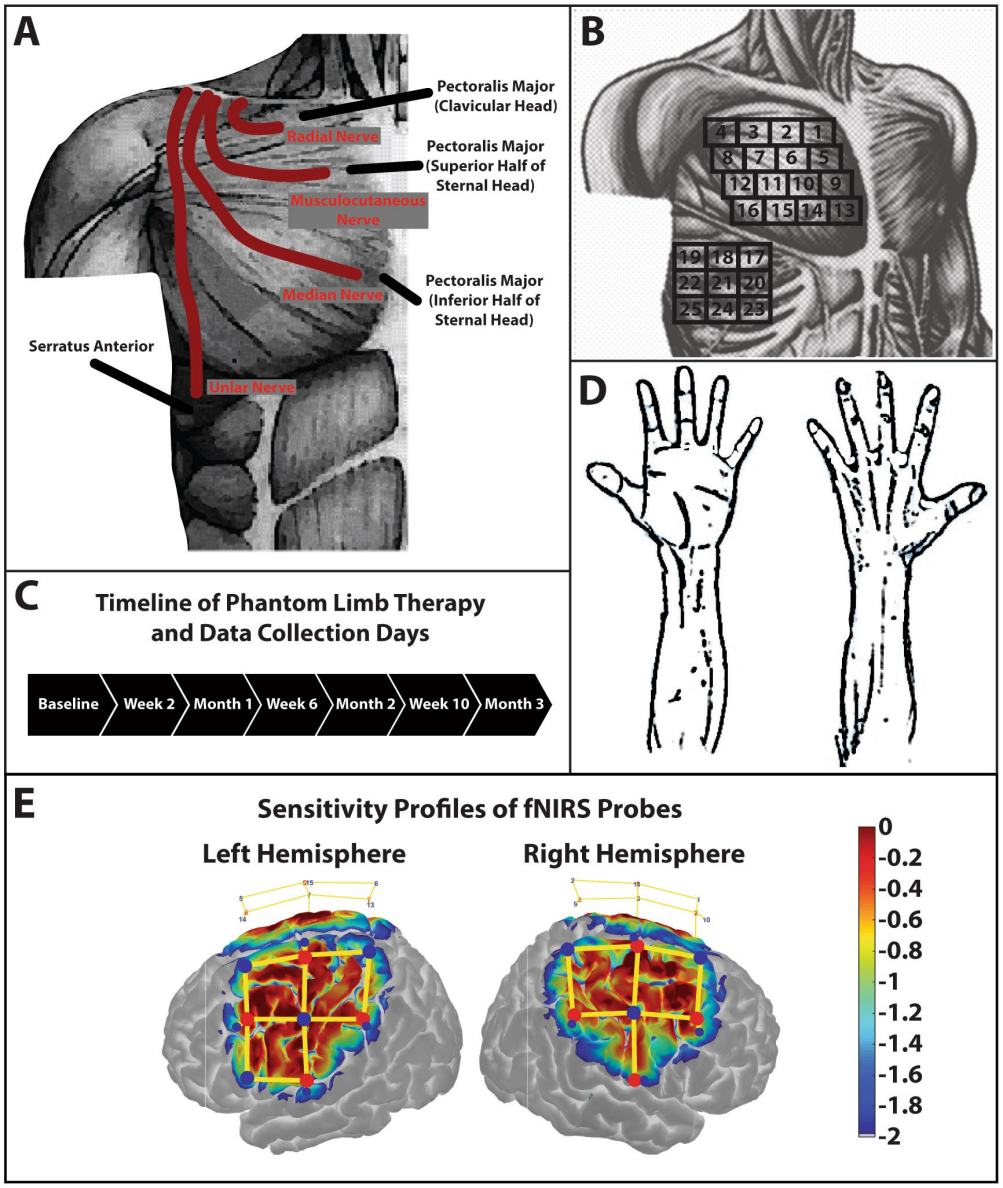
Schematic of TMR procedures and sensory testing. **(A)** Schematic of the case participant presenting with a short trans-humeral amputation after a targeted muscle reinnervation surgery. The radial nerve was transferred to the nerve branch of the clavicular head of the pectoralis major. The musculocutaneous nerve was transferred to the nerve branch that innervated the superior half of the sternal head of the pectoralis major. The median nerve was transferred to the nerve branch that innervated the inferior half of the sternal head of the pectoralis major. Lastly, the ulnar nerve was transferred to the lateral thoracic nerve which innervated the serratus anterior. Subcutaneous fat was not removed secondary to the patient’s low body fat content. **(B)** The grid used to palpate the reinnervation sites. This grid along with anatomical landmarks clavical, sternum, axilla, and mamilla were used to reduce variability and increase consistency of palpation sites between data collection days. **(C)** Phantom limb therapy occurred every day for 3-months. Data collection sessions were conducted every 2-weeks over the 3-month period. **(D)** Palmar and dorsal side of the hand. The subject pointed to the area of the hand or arm where he felt the sensory sensation when a stimulus was produced by palpating the reinnervation sites. **(E)** Sensitivity profile of the probe over the sensorimotor cortex used in this study. Log sensitivity index values closer to zero (red) reflect greater sensitivity for acquiring signals in that region (Aasted et al., 2015). Image created using AtlasViewer (v2.12.4). The location of the detectors (blue), the sources (red), and the channels (yellow lines) are shown for the subject.

The case participant was a 21-year-old male manual-laborer who sustained a traumatic amputation to his dominant right arm which resulted in a high transhumeral amputation above the level of the deltoid and pectoralis insertion. Upon initial presentation, the subject had a traumatic amputation with rapid bleeding from the brachial artery. This was suture ligated along with the brachial plexus in the acute trauma resuscitation. This functionally left the subject with a shoulder disarticulation. The subject underwent a TMR procedure after the damage control procedure through a distinct surgical site proximal to the traumatic amputation where the four main nerves of the brachial plexus after the divergence of the axillary nerve were transferred to various nerves of chest wall muscles (Figure 1A).

The subject performed the phantom limb therapy every day at home for a total of 3 months and returned to the lab for data collections every 2 weeks (Figure 1C). The subject signed a medical record information release (HIPAA) form to give researchers access to his medical information. Written and informed consent was provided by the subject to participation in this study and for the publication of this case report (including all data/images), which was approved by the University of Nebraska at Omaha Review Board.

### 2.2. Three month phantom limb therapy

The subject began phantom limb therapy 1-year post-amputation and TMR surgery. The movements chosen for the phantom limb therapy were derived from the original function of the rerouted nerves and the typical movements needed for a potential prosthetic arm in the future: (1) opening the hand, (2) closing the hand, (3) wrist flexion, (4) wrist extension, (5) elbow flexion, and elbow extension. There were three phases to the phantom limb therapy: (1) Perform movements with the intact limb only, (2) Perform the movements with the phantom and intact limbs together, and (3) Perform the movements with the phantom limb only. The subject was instructed to perform each movement 20 times during all phases for a total of 360 movements performed each day. The number of movements performed were chosen from previous use-dependent (Nudo et al., 1996) and prolonged motor imagery (Rozand et al., 2016) studies. After Week 10, the subject was instructed to include two additional movements: (1) Wrist Pronation and (2) Wrist Supination, as it was discovered that these movements could activate the musculocutaneous nerve, which had previously shown minimal to no activity. With the addition of these two wrist movements, the subject performed a total of 480 movements each day, during the final 2 weeks of phantom limb therapy.

### 2.3. Data collection sessions

#### 2.3.1. Sensory mapping

Sensory mapping data were collected by palpating in a grid orientation (Figure 1B) over the pectoralis major and serratus anterior muscles to increase reproducibility between sessions. The subject would then point to the drawing of the hand (Figure 1D) or on the researcher’s hand or arm where he felt the sensation.

#### 2.3.2. Maximum voluntary contractions

Since the subject had no limb to press against a dynamometer, the subject was instructed to produce maximum voluntary contractions (MVCs) to the best of his ability by flexing or squeezing as hard as possible. Each MVC was performed for 10 s followed by 30 s of rest for a total of 3 trials. The following MVCs were performed: biceps flexion, thumb flexion, finger adduction, triceps flexion, and power grip (i.e., squeezing the hand into a fist).

#### 2.3.3. Gross manual dexterity task

The Box and Block (B&B) Test has been suggested as a measure of unilateral gross manual dexterity (Mathiowetz et al., 1985, 1986). The B&B Test followed the procedures outlined in previous experiments (Zuniga et al., 2019; Borrell et al., 2021). When using the phantom limb, the subject was instructed to imaging each step of picking up the block which included the feeling of picking up a block as well as transferring the block across the divider and releasing the block into the adjacent box.

### 2.4. Functional near-infrared spectroscopy

Functional near-infrared spectroscopy (fNIRS) task data were collected and analyzed using similar techniques from a previous case study (Borrell et al., 2021). The main difference in this study is the inclusion of short separation detectors (~8 mm distance), which were used to filter out physiological noise from the scalp (Zhang et al., 2021). The cap was positioned to cover the sensorimotor area (Figure 1E). Task data were analyzed using the Homer3 (v1.26) Toolbox (Huppert et al., 2009; Bunpc Homer3, n.d.). Hemodynamic data were reconstructed on atlas anatomy utilizing the AtlasViewer (v2.12.4) Toolbox (Aasted et al., 2015). HbR data were not analyzed or displayed for this study.

Three minutes of pre-task data were collected for resting state functional connectivity (RSFC) analysis. The raw data was then processed and analyzed using the NIRS Brain AnalyzIR toolboxes (Santosa et al., 2018). Data were down sampled to 4 Hz, optical density was estimated, and the Beer–Lambert Law was used to obtain HbO data. Pearson correlation coefficients (*r*) were calculated using the NIRS Toolbox’s built-in ‘connectivity’ module. This connectivity function employs an autoregressive robust correlation function to help reduce the confounding effects of physiological phenomena that can lead to false positive results (Huppert, 2016; Santosa et al., 2017). Each *r*-value quantifies the correlation between the hemodynamic data of two channels, which serves as a surrogate measure for RSFC. After calculating all connectivity values, they were converted to *Z* values using Fisher’s transformation (Fisher, 1915) which normalizes the variance of Pearson correlation coefficients (*r*). A two-tailed Student’s *t*-test was then applied to obtain the *p*-values.

## 3. Results

### 3.1. Qualitative feedback from the TMR subject

#### 3.1.1. Phantom pain

Before therapy, the subject experienced minimal to nonexistent phantom pain (1 on a 10 point scale with 1 = no pain and 10 = very painful) and only occasionally experienced phantom limb sensations, which were not bothersome. At Month 1, phantom pain was reported after performing the phantom movements during therapy (2 on a 10 point scale). This dull pain went away at Month 2 (1 on a 10 point scale) and did not return.

#### 3.1.2. Fatigue

During baseline recordings, the subject became very fatigued (6–7 on a scale of 10 with 1 = no fatigue and 10 = very fatigued), so it was decided by the researchers to end the data collection early. At Week 2, the subject’s fatigue drastically improved to a 2–3 on a 10 point scale. At Month 3, the subject reported that he felt “perfectly fine” with no fatigue at all (1 on a 10 point scale).

#### 3.1.3. Limb synchronization

During baseline, the subject first noted that the phantom movements “matched pretty well” (9 on a 10 point scale with 1 = limbs not synchronized at all and 10 = limbs synchronized very well). However, at Week 2, the phantom hand was slower during hand opening only (7.5 on a 10 point scale) and that the other movements still “matched pretty well” (9 on a 10 point scale). At Month 3, the subject reported an “almost perfect match” between all phantom and intact movements (9–10 on a 10 point scale).

### 3.2. Qualitative sensory reponses

**Figure 2:**
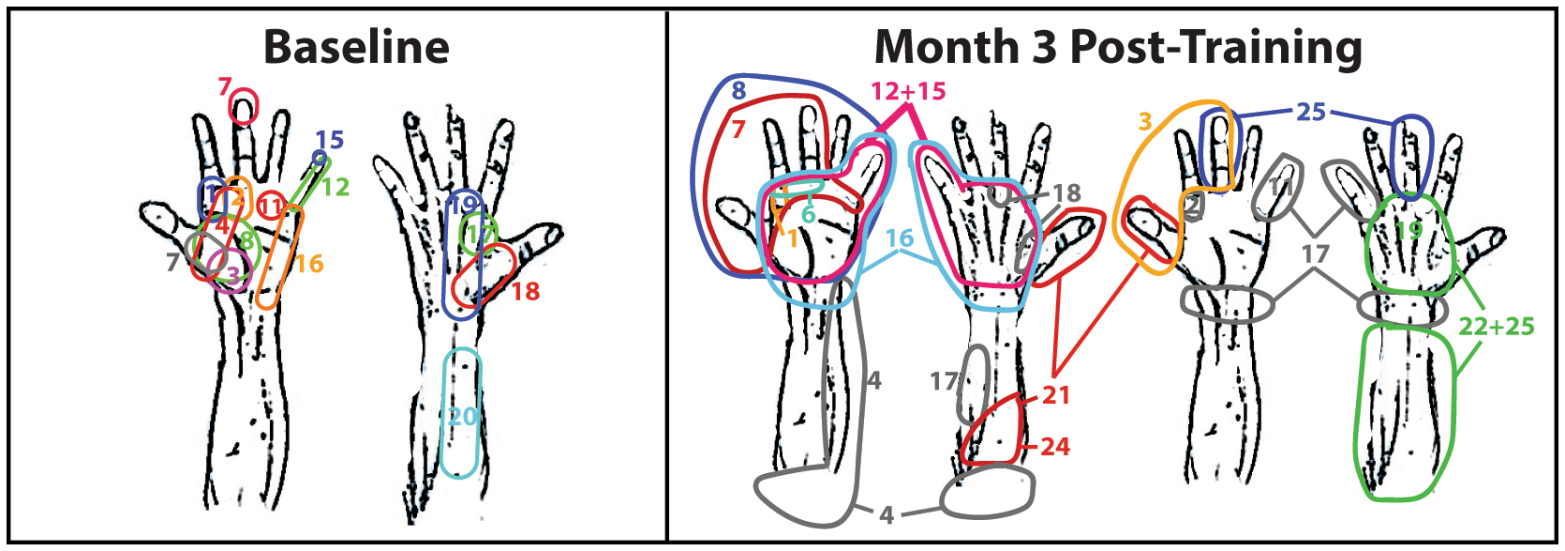
Sensory mapping of the TMR subject. Sensory mapping was conducted in a grid fashion by tapping sites 1—25 over the right major pectoralis and serratus anterior. The numbers correspond to the grid in Figure 1B. The TMR subject indicated the area of the right hand in which he felt the tapping by either pointing at the hand diagram, pointing at the researcher’s hand, or describing the sensation. The color-coding is only meant to differentiate the site numbers in the event they overlap and only corresponds to the adjacent number with the same color. Color-coding is not consistent between weeks of therapy nor between hands in the same week of therapy.

During baseline, 13 of the 25 sensory sites produced no reponses during phantom limb movements. The sensory sites that did produce a response were qualitatively determined as minimal and vastly overlapping (Figure 2). At Month 3, the sensory sites produced large areas of sensory reponses, and the number of no response sensory responses decreased to eight sites. Active sensory sites started to qualitatively expand and separate (Figure 2).

### 3.3. Cortical responses during movement tasks

**Figure 3:**
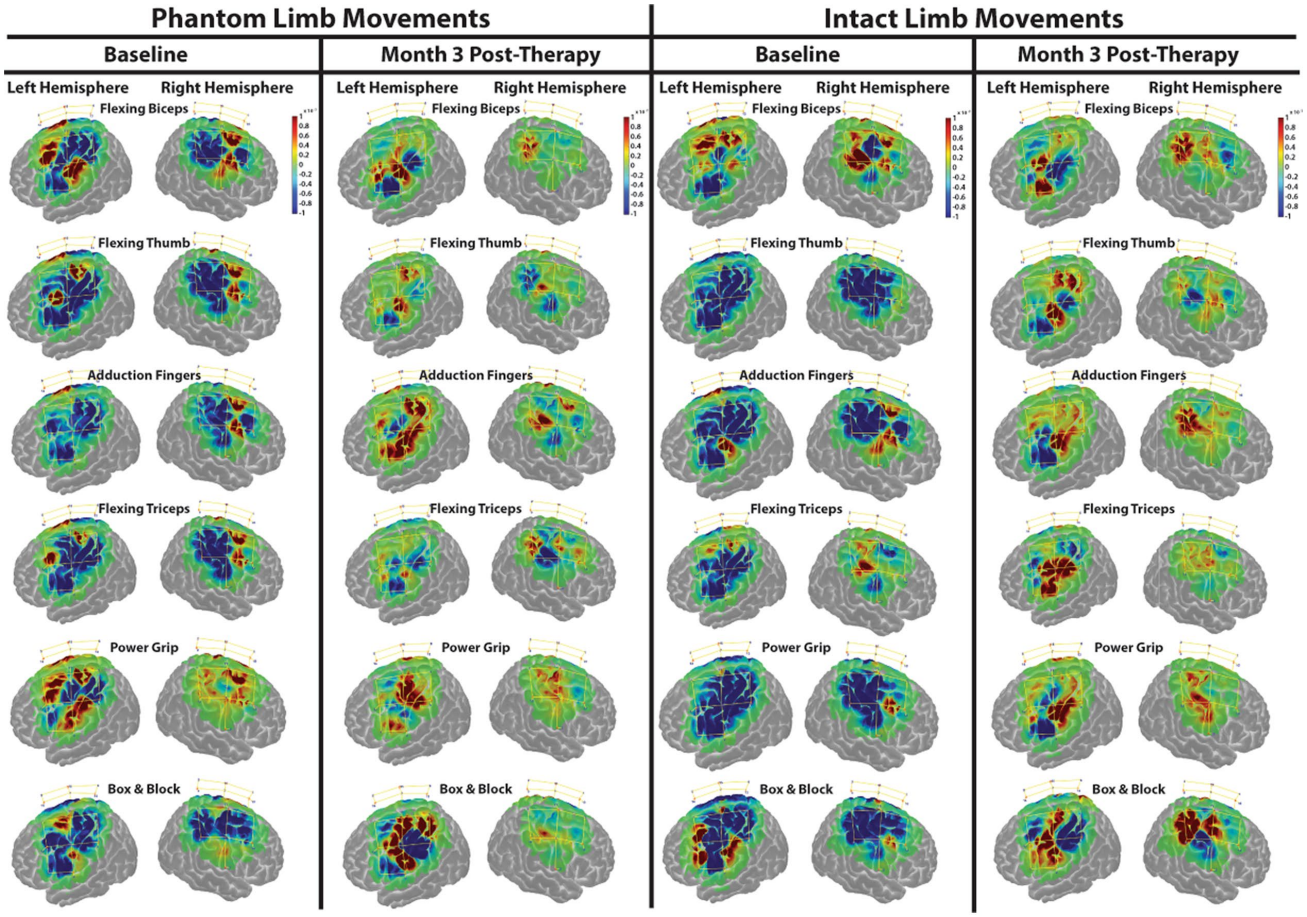
Functional near-infrared spectroscopy (FNIRS) spatial activty maps during phantom and intact limb movements. Change in HbO, as compared to the expected modeled response, is shown for each channel (yellow lines). An increased HbO response from the expected response is highlighted in red while a decreased HbO reponse is highlighted in blue. The change in HbO is centered on zero where zero is the expected modeled response. Only changes in HbO greater than a threshold of *p* < 0.01 were plotted. The color bar indicates the scale of the concentration change on the logarithmic scale. Red dots are source optodes, and blue dots are detector optodes. Figures were created using AtlasViewer.

During baseline, changes in HbO were drastically different from the expected response model (measured around zero and represented in green) during both intact and phantom limb movements. During these movements, signficant decreases in HbO, represented as deep blue in Figure 3, appeared to dominate over the smaller areas of signficantly increased HbO levels, represented as deep red, that occurred more in the premotor and primary motor areas. Increases and decreases in HbO were present in both hemispheres indicating a bilateral response during phantom and intact movements.

At Month 3 (Figure 3), changes in HbO became more balanced especially in the right, afferented hemisphere during both intact and phantom movements. The drastic decreases in HbO appeared to diminish and the drastic increases in HbO occurred more in the primary motor area of the left, deafferented hemisphere during both intact and phantom movements.

### 3.4. Resting state functional connectivity

**Figure 4:**
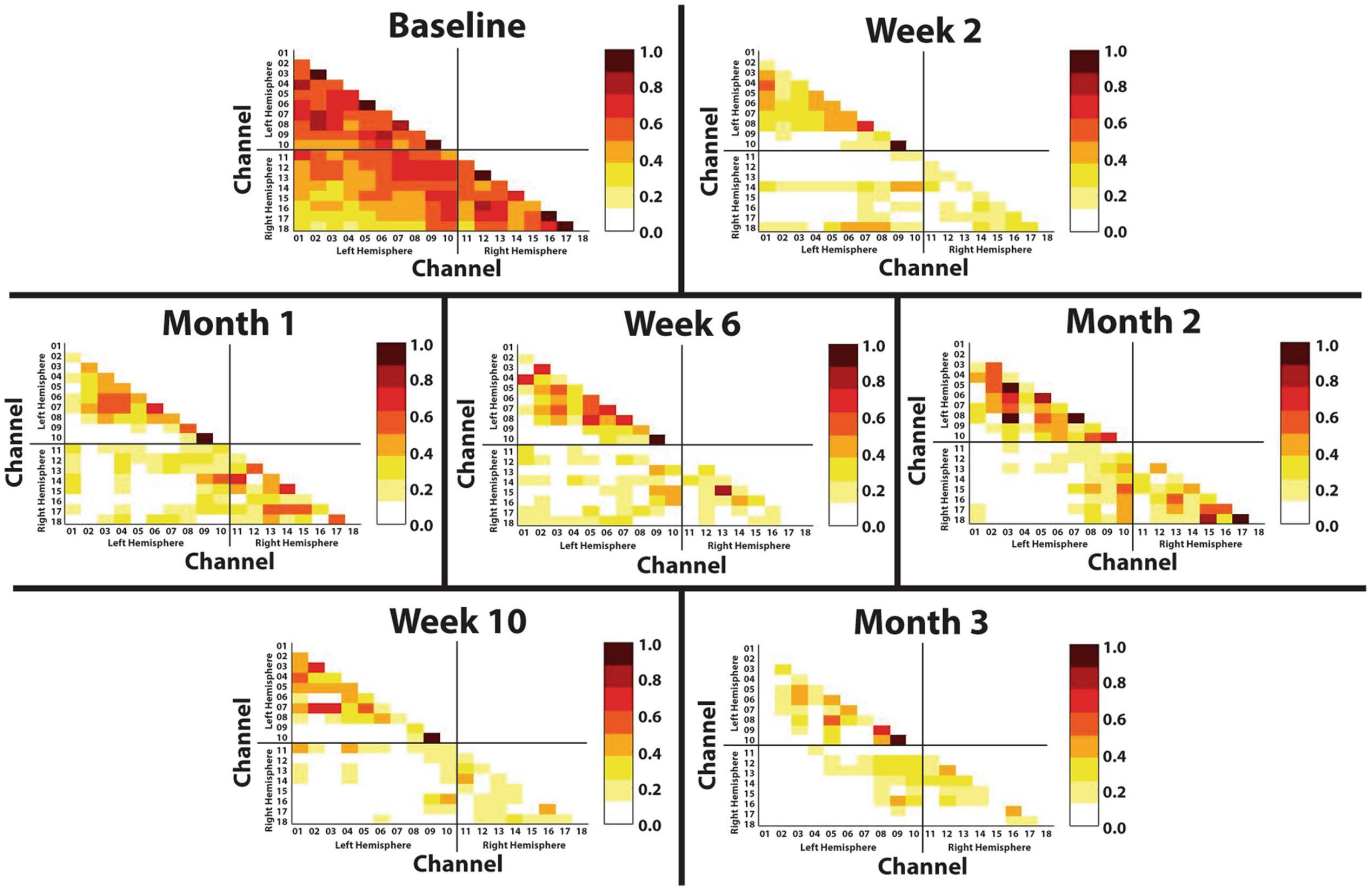
Resting state functional connectivity throughout 3-months of phantom limb therapy. The *Z*-score of the correlation matrix for HbO activation from all the channels recorded during a 3-min resting period. Channels 1–10 represent the left, deafferented hemisphere in the upper left quadrant, channels 11–18 represent the right hemisphere in the lower right quadrant, and the channels from both hemispheres in the lower left quadrant represent the interhemispheric connectivity.

The Fisher transformed matricies are shown in Figure 4. During baseline, the primary motor (M1), premotor (PMC), and sensory (S1) corticies within both the left hemisphere (left M1 sum of *Z*-scores = 9.50; left PMC sum of *Z*-scores = 6.45; left S1 sum of *Z*-scores = 2.42) and right hemisphere (right M1 sum of *Z*-scores = 6.54; right PMC sum of *Z*-scores = 1.79; right S1 sum of *Z*-scores = 6.78) are shown to have a strong correlation within the respective hemispheres (left hemisphere sum of *Z*-scores = 28.08; right hemisphere sum of *Z*-scores = 16.67), indicating strong intra-hemispheric connections. Additionally, these same sensorimotor regions are shown to have a strong correlation between hemispheres, indicating strong inter-hemispheric connections (M1 sum of *Z*-scores = 15.50; PMC sum of *Z*-scores = 7.87; S1 sum of *Z*-scores = 4.28; All Channels sum of *Z*-scores = 39.05). By Month 3, correlation strength was drastically decreased as compared to Baseline in both inter-hemispheric (M1 sum of *Z*-scores = 3.21; PMC sum of *Z*-scores = 1.49; S1 sum of *Z*-scores = 0.92; All Channels sum of *Z*-scores = 6.58) and intra-hemispheric (left M1 sum of *Z*-scores = 4.55; left PMC sum of *Z*-scores = 1.43; left S1 sum of *Z*-scores = 0.38; right M1 sum of *Z*-scores = 1.34; right PMC sum of *Z*-scores = 0.63; right S1 sum of *Z*-scores = 2.01) channels. Interestingly, channels 09 and 10 within the left, deafferented M1 did not change and continued to show a strong correlation.

## 4. Discussion

After 3-months of phantom limb therapy that was derived specifically for this case subject, the results displayed signficant changes of cortial activity measured as changes in HbO levels during various tasks and decreased correlation strength between inter- and intra-hemisphereic connections during rest. In addition, the subject experienced reduced fatigue, a fluctuation in phantom pain, improved limb syncronization, and increased sensory sensation.

To our knowledge, this is the first study to describe the sensory outcomes in this format and after TMR surgery. The main limitation of this case study is that data was collected in only one subject without comparisons to a non-amputee, control subject nor to a non-TMR amputee.

Currently, it is recommended that therapy begin immediately after amputation and TMR surgery or right after the tissue has had time to adequately heal after surgery, depending on the condition of the patient. This timeframe did not occur in this case study due to socio-economical barriers that hindered the subject. The authors highly recommend that building relationships between clinicians and researchers become the priority in order to (1) reduce the time between surgery and the onset of therapy and (2) to break down any barriers of access that may hinder a patient. Nonetheless, the results of this study may suggest that a later onset of therapy may still provide benefits to TMR amputees well after their initial amputation and TMR surgery.

It has been shown that the later stage of learning is slow and may take several sessions (or weeks) of practice (Nudo et al., 1996; Karni et al., 1998). As training progresses, Nudo et al. (1996) and Karni et al. (1998) have shown that motor performance becomes fluent and less attention is required to perform the movement, reflecting implicit learning. Our results agree with this notion as shown by more balanced levels of HbO, decreased inter- and intra-hemispheric correlation strength shown during rest, and a qualitative description from the subject that the phantom movements became easier to perform and more fluid which was supported by reduced fatigue as time progressed. Interestingly, the subject reported at the beginning of therapy that the phantom limb felt abstract, mixed-up, and disorganized. At the end of therapy, the subject reported a connected, pieced together phantom limb which he could easily and smoothly move in space. The subject also reported that “my phantom limb feels part of me once again.” This discription and the overall results suggest an improved cortical efficiency of the sensorimotor network observed during phantom and intact limb movements.

### 4.1. Consistency found after amputation

It has been shown that hand movement representations survive in the deafferented cortex of ampuees (Reilly et al., 2006) even when a phantom limb sensation cannot be produced (Mercier et al., 2006). Similarly, it has been reported that motor and sensory representations shifted to their estimated originical locations following TMR surgery (Giraux et al., 2001; Chen et al., 2013; Yao et al., 2015). During baseline, the subject produced signficant changes in HbO based on the expected response while using both limbs, which were similarly reported by Kew et al. (1994). However, they only reported signficant increases of blood flow in the deafferented cortex and not in the afferented cortex (Kew et al., 1994). These results may suggest drastic inhibition in areas of signficant decreases in HbO and increased excitability in areas of significant increases of HbO (Cai et al., 2022). It is uncertain if the recorded HbO levels occurred in the original cortical representations; however, this activitiy did occur in both sensorimotor cortices which suggests that the original cortical representations are being utilized during both phantom and intact limb movements. Furthermore, the subject reported minimal phantom limb pain before beginning the phantom limb therapy. Phantom limb pain has been associated with abnormal increases and decreases in cortical plasticity after amputation (Li et al., 2021), which were present during baseline recordings. However, it appeared that the TMR surgery proved successful in reducing phantom limb pain despite these drastic changes in HbO.

### 4.2. Resting state connectivity suggests decoupling

Arm amputation results in immediate deprivation of major inputs and outputs to the sensorimotor network. This eventually leads to unmasking of silenced inputs (Barnes and Finnerty, 2010), which may be the case for the greater inter-and intra-hemispheric connectivity during baseline recordings. This unmasking could be associated with decreases in the neurotransmitter gamma-Aminobutyric acid (GABA) neurons, which has been shown to decrease in adult monkeys after visual deafferentation (Hendry and Jones, 1986) and has been assumed to also occur in humans after amputation (Kew et al., 1994). With greater connectivity, it could easily be assumed that this would result in greater efficiency; however, this may not be the case. Along with greater connectivity during baseline, the subject reported greater fatigue, which is an indication of higher metabolic cost (Bassett et al., 2009). Bassett et al. previously showed in amputees that the missing hand cortex becomes decoupled (i.e., lower levels of functional coupling) from the sensorimotor network and coupled with the default mode network over time. However, it has been shown that amputees without TMR surgery still have strong representations in the sensorimotor network (Ding et al., 2020). Thus, our baseline resting state fNIRS data may suggest that the missing hand cortex may still be coupled to the sensorimotor network.

The decreased correlation values over time suggest that the phantom limb therapy may have decoupled the missing limb from the sensorimotor network, which was suggested to happen over time in non-TMR amputees (Makin et al., 2015). However, it is uncertain if the missing limb became coupled with other areas of the brain. For example, the missing limb may have coupled with the default mode network (Bolwerk et al., 2013) or to sub-cortical networks which are not feasible with this fNIRS set-up. However, due to the TMR surgery which created new closed loops to the chest muscles, it is more likely that the phantom limb representation may have coupled with the trunk representation of the brain. Further evidence is needed to support this claim.

### 4.3. Future considerations of phantom limb therapy

The overall results suggest that all the benefits reported stem from the cortical changes seen in the fNIRS data, which we term as cortical efficiency. These results suggest that the current protocol of phantom limb therapy provided a benefit to the patient. It is difficult to pinpoint which part of the protocol produced the greatest benefit; however, we recommend that various phantom limb movements should be performed based on the level of amputation. This recommendation follows the mechanisms underlying use-dependent plasticity (Nudo et al., 1996; Makin et al., 2013; Raffin and Siebner, 2019). Future implementations of this protocol may consider including additional phantom limb movements or more complex movements as therapy progresses. The goal of modifying the protocol over time would be to train the phantom limb further to perform complex movements in an efficient and fluid fashion.

## 5. Conclusion

The present case study demonstrated that phantom limb therapy performed daily over a 3-month period produced signficant changes of cortial activity, decreased correlation strength between intra- and inter-hemispheric channels, and signficant sensory expansion in a TMR amputee. These cortical improvements directly benefited the TMR amputee by drastically reducing fatigue, improving limb synchronization, and causing a fluctuation in pain that returned to a non-existent state. These combined results suggest an improved cortical efficiency of the sensorimotor network. Furthermore, the strategies behind this phantom limb therapy can be translated to different types of amputations and different reinnervated nerves. As we strive for a humanlike prosthetic limb that restores effortless functionally, we have demonstrated the positive effects of a phantom limb therapy paradigm that could help amputees reduce the training time needed to use a myoelectric device, which will potentially improve strategies used in daily life.

## Data availability statement

The raw data supporting the conclusions of this article will be made available by the authors, without undue reservation.

## Ethics statement

The studies involving human participants were reviewed and approved by the University of Nebraska at Omaha (UNMC) Institutional Review Board (IRB). The patients/participants provided their written informed consent to participate in this study. Written informed consent was obtained from the subject for the publication of any potentially identifiable images or data included in this article.

## Funding

This case study was funded by funded by the National Institute of Neurological Disorders and Stroke of the National Institutes of Health under Award Number [R01 NS114282] along with funding from the Biomedical Rehabilitation and Manufacturing Initiative (BRMI).

## Author contributions

JB conceived and designed the research, drafted the manuscript. AL and SF performed the amputation and TMR surgery. JB, AM, CC, KF, AD’O, and ZG collected the data. JB and AM analyzed the data. JB, AM, AL, SF, and JZ interpreted the results of experiments. JB and AM prepared the figures. JB, AM, and JZ edited and revised manuscript and approved the final version of the manuscript. All authors contributed to the article and approved the submitted version.

## Conflict of interest

The authors declare that the research was conducted in the absence of any commercial or financial relationships that could be construed as a potential conflict of interest.

## Publisher’s note

All claims expressed in this article are solely those of the authors and do not necessarily represent those of their affiliated organizations, or those of the publisher, the editors and the reviewers. Any product that may be evaluated in this article, or claim that may be made by its manufacturer, is not guaranteed or endorsed by the publisher.
